# PERSON-CENTERED COMMUNICATION AND APATHY IN NURSING HOME RESIDENTS WITH DEMENTIA

**DOI:** 10.1093/geroni/igad104.2295

**Published:** 2023-12-21

**Authors:** Ying-Ling Jao, Yo-Jen Liao, Diane Berish, Jacqueline Mogle, Helen Kales, Marie Boltz

**Affiliations:** Pennsylvania State University, State College, Pennsylvania, United States; Penn State University, State College, Pennsylvania, United States; The Pennsylvania State University, University Park, Pennsylvania, United States; Clemson University, Clemson, South Carolina, United States; University of California, Davis Health, Davis, California, United States; Pennsylvania State University, State College, Pennsylvania, United States

## Abstract

Apathy is prevalent in dementia and is associated with poor health outcomes. Communication is fundamental for daily caregiving activities. Person-centered communication has positively impacted some neurobehavioral symptoms, but its impact on apathy remains unclear. This video observation study examined the association between healthcare workers’ use of person-centered communication and apathy in nursing home residents with dementia. Three videos, three to ten minutes each, were recorded for each resident to capture daily caregiving interactions. Healthcare workers’ communication was transcribed and segmented into phrases. Communication was coded phrase-by-phrase via video observations using the Person-Centered Behavioral Inventory. Communication was categorized as none, person-centered, non-person-centered, or others. Apathy was coded second-by-second via video observations using the Person-Environment Apathy Rating. Concurrent correlations and ANOVA were used for analysis. A total of 17 residents with dementia and apathy and 21 healthcare workers (e.g., certified nurse assistants, activity staff, and nurses) were recruited from three nursing homes. Results showed that caregiver communication approach had widely mixed correlations with second-by-second apathy scores across all videos (averaged r=0.09, range: r=-0.18 to r=0.54). Further ANOVA analysis showed that using person-centered communication, as compared to other communication approaches, had mixed associations with apathy; 19.6% showed significantly lower apathy, 35.7% showed significantly higher apathy, and 44.6% showed no significant associations. Overall, healthcare workers’ communication approach can potentially impact apathy for residents with dementia. Future research that examines the sequential associations between communication and apathy is needed to guide intervention development to reduce apathy and improve dementia care in nursing homes.

